# Overexpression of miR156 in switchgrass (*Panicum virgatum* L.) results in various morphological alterations and leads to improved biomass production

**DOI:** 10.1111/j.1467-7652.2011.00677.x

**Published:** 2012-05

**Authors:** Chunxiang Fu, Ramanjulu Sunkar, Chuanen Zhou, Hui Shen, Ji-Yi Zhang, Jessica Matts, Jennifer Wolf, David G J Mann, C Neal Stewart, Yuhong Tang, Zeng-Yu Wang

**Affiliations:** 1 Forage Improvement Division, The Samuel Roberts Noble FoundationArdmore, OK, USA; 2 Department of Biochemistry and Molecular Biology, Oklahoma State UniversityStillwater, OK, USA; 3 Plant Biology Division, The Samuel Roberts Noble FoundationArdmore, OK, USA; 4 BioEnergy Science CenterOak Ridge, TN, USA; 5 Department of Plant Sciences, University of TennesseeKnoxville, TN, USA

**Keywords:** biofuel crop, biomass, miR156, microRNA, *Panicum virgatum*, transgenic switchgrass

## Abstract

Switchgrass (*Panicum virgatum* L.) has been developed into a dedicated herbaceous bioenergy crop. Biomass yield is a major target trait for genetic improvement of switchgrass. microRNAs have emerged as a prominent class of gene regulatory factors that has the potential to improve complex traits such as biomass yield. A miR156b precursor was overexpressed in switchgrass. The effects of miR156 overexpression on SQUAMOSA PROMOTER BINDING PROTEIN LIKE (SPL) genes were revealed by microarray and quantitative RT-PCR analyses. Morphological alterations, biomass yield, saccharification efficiency and forage digestibility of the transgenic plants were characterized. miR156 controls apical dominance and floral transition in switchgrass by suppressing its target *SPL* genes. Relatively low levels of miR156 overexpression were sufficient to increase biomass yield while producing plants with normal flowering time. Moderate levels of miR156 led to improved biomass but the plants were non-flowering. These two groups of plants produced 58%–101% more biomass yield compared with the control. However, high miR156 levels resulted in severely stunted growth. The degree of morphological alterations of the transgenic switchgrass depends on miR156 level. Compared with floral transition, a lower miR156 level is required to disrupt apical dominance. The improvement in biomass yield was mainly because of the increase in tiller number. Targeted overexpression of miR156 also improved solubilized sugar yield and forage digestibility, and offered an effective approach for transgene containment.

## Introduction

Plants store energy through the process of photosynthesis in their biomass and provide a sustainable source for energy conversion. Fossil fuels were formed by prehistoric biomass in the form of non-renewable resources. These finite fossil fuel reserves and the impact of fossil fuel emissions on the atmosphere have caused serious public concerns (Somerville *et al.*, 2010). In recent years, biofuels have been exploited as an alternative energy source to meet the growing energy demands worldwide (Henry, 2010). Lignocellulosic biofuels can be produced from non-food crops such as switchgrass, Miscanthus, willow and poplar (Yuan *et al.*, 2008; Somerville *et al.*, 2010). The polysaccharides stored in cellulosic biomass can be converted to biofuels through microbial fermentation, pyrolysis or gasification. Switchgrass has been identified as a dedicated bioenergy crop by the U.S. Department of Energy. It is a C4, warm-season perennial bunchgrass with the potential to be grown on marginal lands (McLaughlin *et al.*, 2006; Schmer *et al.*, 2008). For all bioconversion processes, dry matter biomass yield is a major objective for switchgrass feedstock improvement (Bouton, 2007).

Genetic engineering is expected to play an important role in improving the quantity and quality of biomass (Hisano *et al.*, 2009; Li and Qu, 2011). Genetic modification of enzymatic genes or transcription factor genes has led to the generation of transgenic switchgrass with improved sugar release and processing properties (Fu *et al.*, 2011a,b; Saathoff *et al.*, 2011; Xu *et al.*, 2011). However, little progress has been made in improving biomass yield of bioenergy crops through genetic engineering approaches. Biomass yield is a highly complex trait and various approaches have been tested in model systems to improve this important trait. Improvement in biomass has been reported by altering gibberellin metabolism in tobacco (Biemelt *et al.*, 2004), modulation of brassinosteroid biosynthesis in rice (Sakamoto *et al.*, 2006), diverting chloroplastic glycolate from photorespiration in Arabidopsis (Kebeish *et al.*, 2007), increasing phosphoribosylpyrophosphate synthetase activity in Arabidopsis and tobacco (Koslowsky *et al.*, 2008), manipulation of WRKY transcription factor in Arabidopsis (Wang *et al.*, 2010) and chloroplast expression of β-glucosidase in tobacco (Jin *et al.*, 2011).

The identification and manipulation of major regulatory genes that govern the expression of a group of downstream genes provide an effective way to improve complex traits. In recent years, miRNAs have emerged as a prominent class of gene regulatory factors. The majority of miRNAs regulate plant growth and development by controlling the levels of transcription factors (Zhang *et al.*, 2006). Plant miR156 is a family of small, non-coding, endogenous RNAs with a relatively high expression level in the juvenile phase of plants. The level of miR156 gradually decreases with plant age (Wu *et al.*, 2009; Matts *et al.*, 2010). The precursor of miR156 forms a stem-loop structure which can be recognized by a Dicer-like protein (DCL1) and processed into 20–21 nt products that bind to its target genes and result in cleavage of mRNA or repression of translation (Park *et al.*, 2002; Chen, 2009).

In plants, most members of the SQUAMOSA PROMOTER BINDING PROTEIN LIKE (SPL) transcription factor family are targeted by miR156 in plants (Rhoades *et al.*, 2002; Xing *et al.*, 2010; Gou *et al.*, 2011). For instance, miR156 targets 11 of the 17 *SPL* genes in Arabidopsis. *SPL*s affect diverse developmental processes such as leaf development, shoot maturation, phase change and flowering in plants. miR156 directly switches off its target *SPL*s at the vegetative phase and regulates the transcription of miR172 through its effects on *SPLs* to promote vegetative phase transition (Poethig, 2009; Wu *et al.*, 2009). Overexpression of miR156 in Arabidopsis repressed the transcript abundance of related *SPL* genes and reduced apical dominance, delayed flowering time, causing dwarfism and increased total leaf numbers and biomass (Schwab *et al.*, 2005). Further research has shown that the Arabidopsis *spl9/spl15* double mutant or 35S:SPL10/11/2-SDX transgenic plants displayed morphological changes observed in the miR156 overexpression plants, albeit less severe (Schwarz *et al.*, 2008; Shikata *et al.*, 2009). The rice genome contains 19 *SPL*s, with 11 of those *SPL*s containing the target sites of OsmiR156. Morphological changes including dwarfism, increased tiller number, late flowering and reduced panicle size were observed in transgenic rice plants overexpressing miR156 (Xie *et al.*, 2006). A polycistronic gene encoding miR156b/c was demonstrated to correspond to the *Corngrass* 1 (*Cg*1) mutation in maize (Chuck *et al.*, 2007). Transgenic maize plants overexpressing miR156b/c showed similar branching and inflorescence phenotype as the transgenic rice plants (Chuck *et al.*, 2007). High levels of miR156 were detected in both transgenic rice and maize; however, the increased number of shoots/tillers in the transgenic plants is accompanied by dwarfism (Xie *et al.*, 2006; Chuck *et al.*, 2007).

With the potential dwarf problem in mind, we hypothesized the severe growth phenotypes were caused by excessive production of miR156. To evaluate the feasibility of improving biomass by manipulation of microRNAs, we generated and analysed a large number of transgenic switchgrass plants. Unlike previous reports, the transgenic plants showed various phenotypes depending on the levels of miR156. We classified the transgenics into three groups based on their morphology and miR156 level. Group I plants had relatively low miR156 levels, normal flowering time, increased tiller number and improved biomass yield. Group II plants showed moderate levels of miR156, severely delayed flowering (non-flowering), moderately reduced plant height, a large increase in tiller numbers and improved biomass yield. Group III plants showed high miR156 levels accompanied by severe dwarfism and increased tiller number, but reduced biomass yield. Group I and group II plants also had significantly increased fermentable sugar production. Furthermore, to detect the most significant molecular changes in the miR156 transgenic lines, microarray analysis was carried out using the transgenic switchgrass plant with the highest level of miR156. Different *SPLs* were identified and their expression pattern was characterized in both control and transgenic plants.

## Results

### Generation of transgenic plants with OsmiR156b overexpression constructs

The OsmiR156b precursor contained a 20 nt-long sequence of rice mature miR156b, which was the same as the switchgrass mature miR156b sequence published by Matts *et al.* (2010). Because no switchgrass miR156 (PvmiRA156) precursors are currently available from the public database, the fragment of the OsmiR156b precursor was employed to generate overexpressed mature PvmiR156 in switchgrass. The pANIC6A-Pre-OsmiR156b construct was generated based on the pANIC6A gateway vector (Mann *et al.*, 2011). A maize ubiquitin gene promoter was placed upstream of the fragment of OsmiR156b precursor (Figure S1a). The selectable marker hygromycin phosphotransferase gene (*hph*) was under the control of the rice actin promoter (Figure S1a). Embryogenic calli were infected with the *Agrobacterium* strain AGL1 carrying the pANIC6A-Pre-OsmiR156b vector, and resistant calli were obtained after hygromycin selection. Green shoots were regenerated after transferring the resistant calli onto regeneration medium. Plantlets with well-developed roots were transplanted to soil.

The greenhouse-grown plants were subjected to PCR screening using *hph* and OsmiR156b precursor-specific primers, respectively. Distinct bands of expected sizes were obtained from forty transgenic events. Southern blot hybridization analysis was used to confirm the transgenic nature of randomly selected lines regenerated from hygromycin-resistant calli. The presence of hybridization signals indicated the transgene was stably integrated into the plant genome (Figure S1b).

### Morphological characterization of transgenic plants

**Figure 1:**
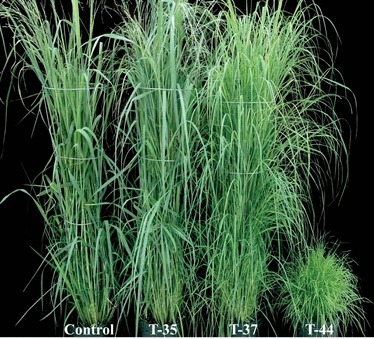
Morphological characterization of transgenic switchgrass plants overexpressing miR156b. Representative plants from each group are shown: T-35 (group I), T-37 (group II) and T-44 (group III).

Twenty-four independent transgenic lines regenerated from the same batch of experiment were employed for morphological analysis in the greenhouse. Based on the characterization of tillers and inflorescences, the transgenic plants were assigned into three groups. Eight of the 24 transgenic lines fell into group I. They showed normal growth and development, but had a significant increase in tiller numbers (1.6- to 2.1-fold of control). Nine transgenic lines were assigned to group II. These plants had a drastic increase in tiller numbers (4.6- to 6.4-fold of control) and normal plant height at vegetative and elongation stages. However, they exhibited short plant height at the reproductive stage because inflorescences were not developed. Seven transgenic lines were assigned to group III. They had a 5.0- to 6.0-fold increase in tiller number but exhibited severely stunted growth. Eight transgenic lines, representing morphological variations among the transgenic plants from groups I to III, were chosen for detailed analyses. Of them, lines T-14, T-35 and T-40 were from Group I; T-27, T-32 and T-37 from Group II; and T-34 and T-44 from Group III. Figure 1 illustrates the morphology of representative plants from each group.

### Expression level of miR156 in transgenic plants

**Figure 2:**
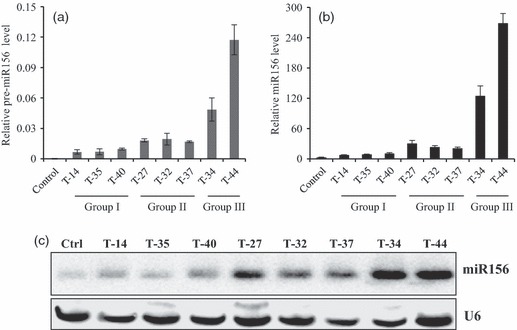
Transcript abundance of pre-miR156b and mature miR156 in transgenic switchgrass plants. (a) Transcript abundance of miR156 precursor in transgenic plants revealed by quantitative RT-PCR. Switchgrass *Ubq1* was used as the reference for normalization. (b) The mature miR156 level of transgenic plants detected and quantified by a highly sensitive quantitative real-time PCR method. miRNA390 was used as the reference for normalization. (c) Small RNA blot analysis of miR156 level in transgenic switchgrass plants. Ctrl: non-transformed plant serving as control. The blot was stripped and rehybridized with oligonucleotide probe complementary to U6 RNA as a loading control.

Vegetative tillers were collected from transgenic plants at V3 stage and used for quantitative RT-PCR analyses. High abundance of the pre-OsmiR156b transcript was detected in transgenic plants, but not in the control (Figure 2a). Furthermore, we examined the level of mature miR156 in transgenic plants by both quantitative RT-PCR and small RNA blot analyses. The transgenic plants had a 3.5- to 128-fold increase in the level of miR156 compared to the control (Figure 2b). Small RNA blot analysis also revealed various mature miR156 levels in the transgenic plants (Figure 2c). The miR156 level corresponded very well to the abundance of pre-OsmiR156b transcript in transgenic lines. Relatively low levels of miR156 were observed in group I plants, moderate levels of miR156 were found in group II lines, and high levels of miR156 were observed in group III plants (Figure 2). The transgenic lines with high miR156 levels (group III) displayed severe morphological alterations. The results revealed that the overexpressed pre-miR156b transcript was successfully processed into mature miR156 and caused distinct morphological changes in transgenic switchgrass plants in a dosage-dependent manner.

### Genes induced by overexpressing miR156 in transgenic plants

Plant miR156 targets members of the *SPL* gene family involved in regulating plant development and growth. The transgenic line T-44 (Figure 1), which showed severe morphological changes, was chosen to investigate the global effects of miR156 overexpression on its downstream genes. Total RNA samples from T-44 and the control were subjected to Affymetrix microarray analysis. Transcript abundance of 2346 probe sets was altered on the chip. There were 1020 probe sets upregulated and 1326 genes downregulated in transgenic plants. Transcript abundance of probe sets representing the *SPL* family was examined. Of 33 probe sets annotated as *SPL* genes, eight probe sets showed 66%–87% reduction in their transcript abundance. Their corresponding cDNA sequences, named as *PvSPL1-8*, were obtained from the switchgrass Unique Transcript Database. Sequence analysis revealed the presence of miR156b target sites in all of the above transcripts. The expression pattern of these unique *SPL* transcripts was analysed by utilizing the switchgrass gene expression atlas. Except for *PvSPL1*, all of the above switchgrass *SPL* genes showed higher transcript abundance in inflorescence and inflorescence meristem than in other organs and tissues.

The expression levels of a large number of other genes involved in diverse function catalogues were altered in transgenic plants. The annotation with Conserved Domain Database (CDD) of probe sets up/downregulated >5-fold was summarized in Table S2. Such large changes in transcript abundance of genes involved in multiple biological processes indicate that overexpression of miR156 triggered a global regulation network in switchgrass.

### Effect of overexpressed miR156 on *SPL* genes in transgenic plants

Phylogenetic comparison based on amino acid sequences of the above eight putative switchgrass SPLs and 19 rice SPLs showed that these switchgrass SPLs belong to three subgroups (Figure S2). Subgroup I includes PvSPL1/OsSPL4/OsSPL11/PvSPL2/OsSPL3/OsSPL12; subgroup II consists of PvSPL3/PvSPL4/PvSPL5/OsSPL14/OsSPL17; and subgroup III comprises PvSPL6/PvSPL7/PvSPL8/OsSPL13.

To validate the effects of miR156 overexpression on *SPLs*, transcript abundance of *PvSPL1*, *PvSPL2*, *PvSPL3* and *PvSPL6* in wild-type and different transgenic lines was analysed by quantitative RT-PCR. In wild-type plants, the transcripts of *PvSPL2*, *PvSPL3* and *PvSPL6* were more abundant in the inflorescence than in other organs. However, *PvSPL1* had a slightly higher transcript level in the immature internode than in the inflorescence (Figure S3).

**Figure 3:**
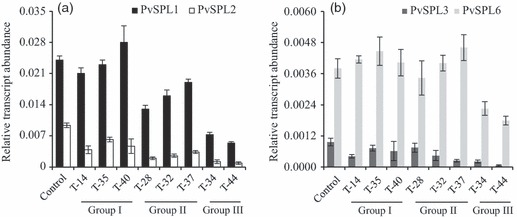
Transcript abundance of putative miR156-targeted *SPL* genes in transgenic switchgrass plants. Transcript abundance of *PvSPL1*, *PvSPL2*, *PvSPL3* and *PvSPL6* was revealed by quantitative RT-PCR. Switchgrass *Ubq1* was used as the reference for normalization.

In the transgenic switchgrass plants, transcript abundance of *PvSPL2* and *PvSPL3* was reduced in all the analysed transgenics compared with the control (Figure 3a,b). The lines with the highest miR156 level had the greatest reduction of *PvSPL2* and *PvSPL3* transcripts. In contrast, the level of *PvSPL6* expression was only reduced in the group III lines (Figure 3b). Interestingly, transcript abundance of *PvSPL1* corresponded well to different groups, with no obvious changes in group I plants, moderate reduction in the lines from group II, and severe reduction in the group III plants (Figure 3a).

### Effects of miR156 expression on plant development and biomass

**Figure 4:**
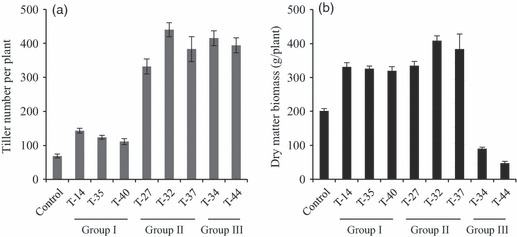
Tiller number (a) and biomass yield (b) of transgenic switchgrass plants. The transgenic and control plants were harvested after 6-month growth in the greenhouse. Values are means ± SE (*n* = 3).

To evaluate the effects of miR156 expression on plant development and biomass, the following traits were measured: dry matter biomass, tiller number, plant height, leaf sheath length, leaf blade length and width, internode length and diameter, internode number and flowering time. The group I transgenic lines T-14, T-35 and T-40 showed apparently normal plant height, flowering time, leaf sheath length, internode number and a 1.6- to 2.1-fold increase in tiller number (Figure 4a and Table 1). Except for line T-14, no difference was observed in leaf blade width, leaf sheath length or internode diameter between the group I lines and the control (Table 1). The significantly increased numbers of tillers of T-14 were sufficient to compensate for the loss in biomass from reduced stem diameter and narrower leaf blade. Dry matter biomass of all the group I transgenic lines showed a 1.58- to1.63-fold increase after 6 months of growth (Figure 4b). The group II transgenic lines T-27, T-32 and T-37 had a 4.8- to 6.4-fold increase in tiller number, but they were significantly shorter than the control, owing to the lack of inflorescences (Figure 4a, Table 1). The plants also had severely reduced internode diameter, leaf blade width and leaf sheath length (Table 1). Because of the large increase in the number of tillers, the transgenic plants had a 1.65- to 2.01-fold increase in dry matter biomass (Figure 4b). The group III lines T-34 and T-44 had the greatest levels of miR156 and exhibited stunted growth. The plant height of the transgenic lines was over 85% shorter than the control. Although the plants showed a 5.7- to 6.0-fold increase in tiller number, they had 85%–88% reduction in internode diameter, 63%–81% reduction in internode length, 76%–80% reduction in leaf sheath length, 56%–68% reduction in leaf blade length and 72%–77% reduction in leaf blade width (Figure 4a and Table 1). The severe dwarfism of group III lines caused more than 50% biomass reduction compared with the control (Figure 4b). The number of internodes of the groups II and III transgenic lines was increased (Table 1), indicating internode number tends to increase when miR156 expression is higher.

**Table 1:** Morphological characterization of transgenic switchgrass plants

	Plant height (cm)	Leaf blade length (cm)	Leaf blade width (cm)	Leaf sheath length (cm)	Internode length (cm)	Internode diameter (mm)	Rang of internode number	Flowering time (day)
Control	242.3 ± 8.6	49.4 ± 3.7	1.24 ± 0.10	18.4 ± 1.6	21.3 ± 1.5	3.76 ± 0.23	4–6	92 ± 2
T0-14	269.5 ± 10.6	41.0 ± 0.6	0.98 ± 0.05*	12.8 ± 0.4*	21.5 ± 1.9	2.63 ± 0.12**	5–6	94 ± 2
T0-35	232.8 ± 9.2	53.0 ± 4.8	1.06 ± 0.05	18.8 ± 2.9	23.1 ± 3.4	3.99 ± 0.18	4–6	93 ± 3
T0-40	240.6 ± 10.1	52.0 ± 4.7	1.02 ± 0.06	19.0 ± 1.9	21.8 ± 1.4	4.05 ± 0.07	4–6	93 ± 2
T0-27	108.8 ± 5.2**	43.0 ± 1.1	0.53 ± 0.03**	11.6 ± 0.5**	20.6 ± 1.1	1.50 ± 0.12**	7–8	>360
T0-32	117.3 ± 3.7**	43.0 ± 2.9	0.65 ± 0.08**	12.2 ± 0.5*	19.5 ± 1.7	1.70 ± 0.18**	6–8	>360
T0-37	110.5 ± 3.5**	41.4 ± 1.0	0.52 ± 0.03**	12.6 ± 0.9*	19.6 ± 0.6	1.57 ± 0.04**	7–8	>360
T0-34	36.0 ± 2.5**	22.0 ± 1.4**	0.35 ± 0.02**	4.4 ± 0.2**	7.8 ± 0.3**	0.58 ± 0.12**	7–8	>360
T0-44	27.5 ± 1.9**	15.6 ± 0.5**	0.29 ± 0.02**	3.7 ± 0.3**	4.0 ± 0.4**	0.44 ± 0.05**	6–8	>360

* Plant height of switchgrass was measured after 6-month growth in the greenhouse. Five-month-old tillers were used to measure internode length (internode 3), internode diameter (internode 3), internode number, leaf sheath length, leaf blade length and width. Five tillers were measured for each replicate. Value are mean ± SE (*n* = 3). One or two asterisks indicate significance corresponding to *P* < 0.05 or 0.01 (one way ANOVA, Dunnett’s test).

### Sugar yield by enzymatic hydrolysis of transgenic plants

**Figure 5:**
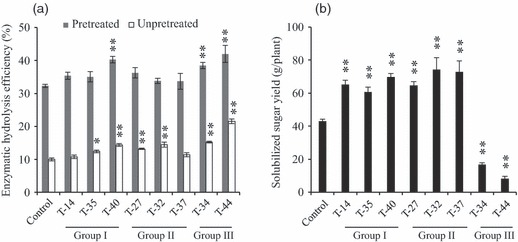
Saccharification efficiency (a) and solubilized sugar yield (b) of transgenic switchgrass plants. Values are means ± SE (*n* = 3). One or two asterisks indicate significance corresponding to *P* < 0.05 or 0.01 (one way ANOVA, Dunnett’s test).

Switchgrass biomass is highly recalcitrant to saccharification of its cell wall carbohydrates. Direct enzymatic hydrolysis only released approximately 10% of total carbohydrates from mature tillers of wild-type switchgrass. Pretreatment with diluted acid allows for removal of most hemicellulose and some of the lignin from lignocellulosic biomass and increases the accessibility of cellulose to cellulase enzymes during saccharification process. Overexpression of miR156 had a significant impact on saccharification efficiency of most transgenic plants without pretreatment, with the exception of T-14 and T-37 which showed no difference compared with the control (Figure 5a). The untreated cell walls of the less recalcitrant transgenic plants had a 24.2%–115.5% increase in saccharification efficiency (Figure 5a). While more sugars were released after diluted acid pretreatment, most of the lines in groups I and II did not show significant changes in saccharification efficiency. A significant increase in saccharification efficiency (19.2%–30.0%) was observed for group III lines after pretreatment (Figure 5a).

The total amount of solubilized sugars produced by acid pretreatment following enzymatic hydrolysis was calculated by combining the biomass and sugar release efficiency. Excluding the lines from group III, transgenic switchgrass lines produced 40.7%–72.3% more solubilized sugars for subsequent fermentation compared with the control (Figure 5b).

### Effects of miR156 expression on forage quality

Forage quality of the transgenic plants was evaluated by measuring *in vitro* true dry matter digestibility (IVTDMD), acid detergent lignin content (ADL) and crude protein content (CP). IVTDMD is an effective indicator of forage digestibility which represents the amount of forage material that can be digested by the rumen of animals. Among the eight transgenic lines tested, six showed a significant increase in IVTDMD. The value of IVTDMD increased from 62.9% in the control to 66.5%–74.7% in the transgenic plants (Table 2). Lignin negatively impacts forage digestibility as well as bioconversion to ethanol. ADL represents the remaining residue that cannot be degraded by sulphuric acid. No significant difference in ADL content was found between the transgenic and control plants, even for the transgenic lines from group III (Table 2). The AcBr method reveals more lignin than the ADL procedure because it includes lignin components that can be solubilized by strong acid. AcBr lignin analysis showed a 15.5%–18.2% decrease in the group III lines. No difference in AcBr lignin content was found in the other transgenic lines (Figure S4).

**Table 2:** Forage quality analysis of transgenic switchgrass plants

	IVTDMD (%)	ADL (mg/g DW)	Crude protein (mg/g DW)	Extractive component (mg/g DW)
Control	62.9 ± 1.4	75.1 ± 3.6	107.7 ± 5.2	281.7 ± 7.5
T-14	62.1 ± 0.4	82.3 ± 0.1	109.2 ± 0.6	283.3 ± 12.0
T-35	67.1 ± 0.1^*^	68.4 ± 0.8	110.0 ± 0.5	296.7 ± 3.3
T-40	64.5 ± 0.1	68.7 ± 1.0	123.5 ± 1.1^*^	266.7 ± 17.7
T-27	67.8 ± 0.1^*^^*^	65.8 ± 1.9	118.7 ± 0.9	310.0 ± 5.8
T-32	66.5 ± 0.2^*^	68.5 ± 0.7	116.5 ± 1.9	296.7 ± 8.8
T-37	66.7 ± 0.3^*^	67.4 ± 0.2	120.5 ± 1.2	280.0 ± 11.6
T-34	70.3 ± 0.2^*^^*^	70.2 ± 1.7	126.8 ± 0.5^*^^*^	350.0 ± 10.0^*^^*^
T-44	74.7 ± 0.3^*^^*^	65.4 ± 2.2	143.3 ± 1.0^*^^*^	353.3 ± 6.8^*^^*^

ADL, acid detergent lignin; IVTDMD, *in vitro* true dry matter digestibility. ^*^The transgenic and control plants were harvested after 6-month growth in the greenhouse. Value are mean ± SE (*n* = 3). One or two asterisks indicate significance corresponding to *P* < 0.05 or 0.01 (one way ANOVA, Dunnett’s test).

Crude protein roughly represents the total amount of protein present in forage. Compared with the control, group III lines had a 17.7%–33.1% increase in CP content. No difference in CP content was found in most of the group I and group II transgenic lines, except T-40, which showed increased CP content (Table 2). Similarly, 24% more non-cell wall components were extracted from the group III lines (Table 2).

## Discussion

Alteration of plant morphology has the potential to improve biomass yield of bioenergy crops (Jakob *et al.*, 2009). Plant transcription factors SPLs directly affect morphology by controlling apical dominance and floral transition. However, functional redundancy of the *SPL* family prevents the application of RNAi disruption or individual mutation (Schwarz *et al.*, 2008; Shikata *et al.*, 2009). miR156 targets most members of the *SPL* family and regulates plant developmental processes caused by *SPL*s. Thus, overexpressing miR156 in switchgrass is a sensible strategy to repress the transcript levels of *SPL*s.

Switchgrass leaf blade and stem had relatively high miR156 levels, while inflorescences accumulated low levels of miR156, consistent with its function in phase transitions from juvenile to adult by targeting SPL transcription factors (Matts *et al.*, 2010). High levels of miR156 in young plants prevent precocious flowering. A decline in miR156 abundance provides a permissive environment for flowering and is paralleled by a rise in SPL levels (Wang *et al.*, 2009). At reproductive stage, seven out of eight switchgrass *SPL* genes analysed showed higher transcript abundance in inflorescences than in other tissues. The only exception was *PvSPL1,* which exhibited relatively high transcript abundance in stems. Further analysis showed that transcript abundance of *PvSPL1* was reduced only in the transgenic lines with a large reduction in internode diameter, implying that *PvSPL1* may play certain roles in stem development (Figure 3a and Table 1). Microarray analysis revealed a severe reduction in *PvSPLs* transcript abundance in the transgenic line with the highest miR156 level. The transcript abundance of switchgrass *SPL* genes was further quantified to study the impact of miR156 on its targets in different transgenic lines. Among the four putative switchgrass *SPL* genes analysed, transcript abundance of three *SPL* genes (*PvSPL1, 2* and *3*) was significantly reduced in the transgenic lines with a 10-fold higher miR156 level. Transcript abundance of one *SPL* gene (*PvSPL6*) was altered only in the lines with a 59-fold higher miR156 level. Furthermore, transcript abundance of *PvSPL1* and *PvSPL*6 was not changed in the lines with moderately increased miR156 level (<6-fold). The results suggest that a threshold miR156 level is associated with its target *SPLs* expression level. The threshold level of miR156 required for alterations of target *SPLs* transcripts increased in the following order: *PvSPL2* and *PvSPL3* > *PvSPL1* > *PvSPL6*. The phenotypes observed in the transgenic switchgrass plants are likely to represent a balanced effect of each individual *SPL* genes.

Overexpression of miR156 caused abnormal development and dwarfism in transgenic rice and maize (Xie *et al.*, 2006; Chuck *et al.*, 2007). These particular transgenic rice and maize plants would seem to have no practical value. Furthermore, biomass characteristics in these transgenic materials were not evaluated. Different from the previous reports, our transgenic switchgrass plants showed various phenotypic changes corresponding to the levels of miR156. Based on plant phenotype and the miR156 level, the transgenic switchgrass plants were sorted into three groups. The group I transgenic lines with relatively low miR156 levels showed both increased tiller numbers and normal growth and development. The 1.6- to 2.1-fold increase in tiller numbers contributed to the 58%–63% increase in biomass yield in these lines. The group II lines had moderate levels of miR156 and showed no sign of flowering after 360 days of growth (Table 1). These plants produced 65%–101% more biomass yield because of a 4.8- to 6.4-fold increase in tiller numbers. Extremely stunted growth was observed in the group III plants with very high levels of miR156. Biomass of group III plants was drastically reduced even though tiller number was increased. Detailed observation revealed that the stunted phenotype was caused by shortened internode length rather than reduced internode number.

Phenotype evaluation coupled with molecular analysis also revealed that miR156 affects tiller number and flowering time in a dose-dependent manner. Based on detailed analysis of eight transgenic plants, it is estimated that switchgrass plants require a threefold increase in miR156 level to disrupt the apical dominance, but require at least a 10-fold higher miR156 level to turn off the floral transition. Thus, it seems genes controlling tiller number were more sensitive to miR156 regulation than those involved in floral transition. The results from miR156 overexpression in rice and maize also indirectly supported this hypothesis, because the transgenic rice and maize plants showed large increases in tiller numbers but only moderately delayed flowering time (Xie *et al.*, 2006; Chuck *et al.*, 2007).

Lignocellulosic biomass is typically highly recalcitrant to bioconversion of its carbohydrates into biofuels (Abramson *et al.*, 2010). Therefore, it is important to assess the effect of miR156 expression on saccharification efficiency and sugar yield of the transgenic biomass. Most of the transgenic lines showed increased saccharification efficiency without pretreatment of the materials. However, the difference in saccharification efficiency between the control and groups I and II plants diminished after pretreatment with diluted acid. Although the reason for such a change is unknown, it is clear that overexpression of miR156 does not have any negative impact on saccharification efficiency. Because of the large increase in biomass yield, the group I and group II transgenic plants produced 40.7%–72.3% more solubilized sugars from their biomass harvested after half year’s growth. Such a large increase in solubilized sugar yield will significantly increase the yield of fermentation products after bioconversion.

In addition to improved sugar yield, many transgenic switchgrass lines showed increased forage digestibility. Because switchgrass can be used as a dual-purpose (bioenergy/forage) crop (Guretzky *et al.*, 2011), development of cultivars with improved biomass yield and increased digestibility could offer more flexibility for farmers.

It is obvious that group I transgenic lines offer a significant potential for genetic improvement of biomass production in switchgrass. Field experiments are needed to confirm the greenhouse data. The non-flowering group II transgenic plants might also prove to be valuable for ultimate deployment because in addition to higher yield and lower recalcitrance, they would enable biocontainment of transgenes. As an outcrossing species, pollen-mediated transgene flow is a major concern for field release of transgenic bioenergy crops (Stewart, 2007; Strauss *et al.*, 2010; Ge *et al.*, 2011). Delayed flowering and complete floral inhibition have been considered desirable target traits for genetic manipulation of grasses (Spangenberg *et al.*, 1998; Jensen *et al.*, 2004; Wang and Ge, 2006). Delay of flowering will minimize the risk of cross pollination between transgenic and non-transgenic materials. Inhibition of flowering will completely avoid the problem of transgene flow in switchgrass. An additional benefit is that non-flowering plants may relocate more carbon to roots instead of reproductive organs, thus increasing carbon sequestration. Furthermore, grass pollen is a major source of allergenic protein, and inhibition of flowering will reduce allergic reactions caused by grass pollen. To date, there has been no report on successful manipulation of flowering time in warm-season grasses. The group II transgenic switchgrass offers a unique opportunity for field testing of the materials for transgene containment and other beneficial traits. Initially, the transgenic lines can be easily propagated by splitting tillers or by the use of an efficient *in vitro* culture procedure (Alexandrova *et al.*, 1996). Future study should involve the development of a seed production system.

Improving biomass yield and reducing biomass recalcitrance are the two critical traits required for the development of next-generation bioenergy crops. Genetic modification of lignin biosynthesis in switchgrass has led to the generation of phenotypically normal plants that have reduced thermal-chemical and enzymatic recalcitrance (Fu *et al.*, 2011a,b; Xu *et al.*, 2011). For example, downregulation of the caffeic acid *O*-methyltransferase (COMT) gene decreases lignin content modestly and increases the ethanol yield by up to 38% using conventional biomass fermentation processes (Fu *et al.*, 2011a). Transgene pyramiding allows for the combination of two or more desirable traits into a novel germplasm. It is anticipated that crossing of the current miR156 overexpression lines with the previous COMT downregulated lines or other lower recalcitrance transgenic events should facilitate the development of superior switchgrass lines for biofuel production.

## Experimental procedures

### Plant materials

A widely used and highly productive lowland-type switchgrass cultivar, Alamo (2n = 4x = 36), was used for genetic transformation and biomass improvement. Switchgrass plants were grown in the greenhouse under a 16-h light photoperiod (6:00 am–10:00 pm) with supplementary lighting supplied by parabolic aluminized reflector lamps (average 390 μE/m^2^/S^1^). The temperature in the greenhouse ranged from 25 to 29 °C (average 26 °C). Plants were watered three times a week and fertilized with Peters 20-10-20 fertilizer (J.R. Peters Inc., Allentown, PA). Primary transgenic plants were propagated by transferring the same numbers of tillers into each pot. Three copies of each line were grown in 3-gallon pots and randomly distributed into three blocks. Border plants were grown around each block. The development of switchgrass in the greenhouse was divided into three vegetative stages (V1, V2 and V3), five elongation stages (E1, E2, E3, E4 and E5) and three reproductive stages (R1, R2 and R3) according to the criteria described by Moore *et al.* (1991).

### Gene constructs and transformation

A 256-bp stem-loop fragment of OsmiR156b precursor was amplified by PCR from the rice genome by using primers Pre-miR156-F and Pre-miR156-R (Table S1) and inserted into pENTR™/D-TOPO® cloning vector (Invitrogen, Chicago, IL). After LR recombination reactions (Invitrogen), the fragment was transferred into the pANIC6A vector (Mann *et al.*, 2011), which carries the selectable marker gene hygromycin phosphotransferase (*hph*). The final binary vector pANIC6A-Pre*-*OsmiR156b was transferred into *Agrobacterium tumefaciens* strain AGL1 using the freezing/heat shock method. Highly embryogenic calli of the switchgrass cultivar Alamo were used for *Agrobacterium*-mediated transformation following the procedure described by Xi *et al.* (2009).

### PCR and Southern blot analysis of transgenic plants

Genomic DNA was isolated from freeze-dried leaf materials of greenhouse-grown plants following the modified 2 × CTAB procedure (Doyle and Doyle, 1987). Transgenic switchgrass was identified by PCR with specific *hph* and pre-miR156 primers (Table S1) using the following conditions: 95 °C for 2 min (one cycle); 94 °C for 30 s, 55 °C for 30 s, 72 °C for 30 s (30 cycles); and 72 °C extension for 10 min. The expected sizes of PCR products are 375 and 256 bp for *hph* and pre-miR156, respectively.

For Southern hybridization analysis, DNA was digested with the restriction enzyme *Nco*I which cleaves twice in the sites outside of the *hph* gene sequence in the binary vector (Figure S1). Twenty micrograms of DNA from each sample was digested overnight and loaded in each lane. The hybridization probe (*hph*) was labelled with digoxigenin (DIG) by PCR (Roche Applied Science, Indianapolis, IN, USA). Gel electrophoresis, DNA blotting and hybridization were carried out following the manufacturer’s instruction manual (DIG High Prime DNA labelling and detection starter kit II). Hybridization signals were detected using the chemiluminescent substrate CSPD-*Star* (Roche Applied Science).

### Quantitative real-time analysis and small RNA blot analysis

Quantitative RT-PCR was performed to analyse transcript abundance of the miR156b precursor in the transgenic plants. Total RNA was extracted from the whole tillers at V3 stage by Tri-Reagent (Invitrogen) and subjected to reverse transcription with Superscript III Kit (Invitrogen) after treatment with TURBO™ DNase I (Ambion, Austin, TX). SYBR Green (Applied Biosystems, Foster City, CA) was used as the reporter dye. The primers used for qRT-PCR are listed in Table S1. The cycle thresholds were determined using ABI PRISM 7900 HT sequence detection system (Applied Biosystems), and the data were normalized using the level of switchgrass *Ubq*1 transcripts (GeneBank accession number: FL899020).

The mature miR156 level was detected and quantified by a highly sensitive real-time looped RT-PCR procedure (Varkonyi-Gasic *et al.*, 2007). The reverse transcription reaction was performed with a miR156 specific stem-loop primer (Table S1). Following stem-loop reverse transcription, miRNA-specific forward primer and a universal reverse primer designed to bind the stem-loop RT primer sequence were used for SYBR Green quantitative RT-PCR. The data were normalized using the level of switchgrass miR390 transcripts.

The mature miR156 level was also detected by small RNA blot analysis (Matts *et al.*, 2010). Twenty micrograms of total RNA was resolved on a denaturing 15% polyacrylamide gel and electrophoretically transferred to Hybond-N+ membranes (GE Healthcare, Piscataway, NJ, USA) followed by cross linking. DNA oligonucleotide complementary to miR156 was end-labelled with γ-32P-ATP using T4 polynucleotide kinase (Invitrogen) and used as a probe. The blot was pre-hybridized for at least 1 h and hybridized overnight at 38 °C using PerfectHyb Plus buffer (Sigma-Aldrich, St. Louis, MO) followed by washing three times at 50 °C. The membrane was exposed to phosphoimager and scanned using a Typhoon scanner. The blot was stripped and re-hybridized with an oligonucleotide probe complementary to U6 RNA as a loading control.

### Microarray analysis of transcript abundance of downstream genes regulated by miR156

Total RNA samples from duplicate biological replicates of the transgenic event T-44 and its corresponding control plants were extracted from whole tillers at V3 stage using Tri-Reagent (Invitrogen), cleaned and concentrated with RNeasy MinElute Cleanup Kit (Qiagen, Valencia, CA). Purified RNA (500 ng) was amplified and labelled using the GeneChip 3’ IVT Express Kit (Affymetrix, Santa Clara, CA) and hybridized to Affymetrix switchgrass cDNA chip containing over 100 000 switchgrass probe sets. Data normalization was conducted by using the robust multi-array average (RMA). Data analysis of differentially expressed probe sets on the chip was performed by associative analysis as described by Dozmorov and Centola (2003).

### Analysis of transcript abundance of *SPL* genes in transgenic switchgrass plants

The sequences of switchgrass *SPL* genes AP13ITG60657 (*PvSPL1*), AP13CTG29191 (*PvSPL2*), AP13ITG56500 (*PvSPL3*), KanlowCTG20060 (*PvSPL4*), KanlowSGLT49238 (*PvSPL5*), KanlowCTG31732 (*PvSPL6*), KanlowCTG41639 (*PvSPL7*) and KanlowCTG07384 (*PvSPL8*) were obtained from the Switchgrass Genomics Database that hosts the switchgrass unique transcript sequences. The corresponding protein sequences were predicted by using the ORF-finder tool on the NCBI website. A phylogenetic tree was generated with MEGA 3.1 software using the neighbour-joining method (Kumar *et al.*, 2004).

The expression pattern of putative switchgrass *SPL* genes was analysed by the switchgrass gene expression atlas. Based on their expression pattern, the sequences of switchgrass *SPL1, 2, 3* and *6* genes were selected for further analysis. The primers were designed to detect transcript abundance of above putative switchgrass *SPL* genes in transgenic plants. Total RNA was isolated from the whole tillers at the V3 stage. Transcript abundance of the putative switchgrass *SPLs* was determined by quantitative RT-PCR. The cycle thresholds were determined using the ABI PRISM 7900 HT sequence detection system (Applied Biosystems), and the data were normalized by using the level of switchgrass *Ubq*1 transcripts.

### Characterization of growth and development of transgenic plants

Transgenic plants were established in the greenhouse. Tiller number, biomass yield, plant height, internode number, internode length, internode diameter, leaf sheath length and leaf blade length and width were measured using 6-month-old transgenic plants. Internode 3 (I3) was used for measuring internode diameter. The leaf and leaf sheath of I3 were used to measure leaf sheath length, leaf blade length and leaf blade width. Biomass harvested from above-ground tissues of 6-month-old plants was dried in an oven at 40 °C for 96 h.

### Measurement of forage quality

The dried switchgrass whole plant samples were ground through a Thomas model 4 Wiley® mill (Thomas Scientific, Swedesboro, NJ, USA) with 1 mm sieve and used for analyses of IVTDMD and ADL. IVTDMD was determined using a near infrared reflectance spectroscopy (NIRS). A sample transport module is configured for NIR reflectance measurements. NIRS analysis was performed using a Foss NIRS 6500 monochromator with a scanning range of 1100–2500 nm (Foss NIR Systems Inc., Silver Spring, MD). Each sample was scanned eight times, and the average spectra were used for calibration. Mathematical and statistical treatments of all spectra were performed with WinISI™ III calibration development software (Foss NIR Systems Inc.). The existing commercial NIRS prediction equations (07GH50-2) developed by NIRS Forage and Feed Testing Consortium were employed to calculate IVTDMD of switchgrass biomass (http://nirsconsortium.org/default.aspx). The precision of NIRS has been assessed by regression analysis of the predicted values and actual determined values. ADL was measured using an ANKOM 200 Fiber Analyzer (ANKOM Technology Corp., Fairport, NY).

### Determination of lignin content

The dried switchgrass whole plant samples were ground through a Wiley® mini mill with a 0.4 mm sieve and used for analyses of lignin content. The extractive-free cell wall samples were prepared as described by Chen and Dixon (2007). The weight of extractive components was measured by the initial dry weight of material minus the dry weight of prepared cell wall residues. The acetyl bromide (AcBr) method described by Hatfield *et al.* (1999) was used to quantify lignin content.

### Determination of cell wall carbohydrate yield and saccharification efficiency

Cell wall residues generated for lignin analysis also were used to analyse total sugar and sugar components released by enzymatic hydrolysis according to previously described procedures (Fu *et al.*, 2011b). For saccharification efficiency analysis, cell wall residues were digested by (i) direct exposure to a cellulase and cellobiase mixture for 72 h (as untreated samples) or (ii) pretreatment with dilute H_2_SO_4_ (1.5%) at 121 °C for 60 min and then exposure to the same enzyme mixture after washing with water (as pretreated samples). Enzymatic saccharification of switchgrass samples was performed following the analytical procedure of the National Renewable Energy Laboratory (LAP-009). Solubilized sugars were analysed spectrophotometrically using the phenol-sulphuric acid assay method (Dubois *et al.*, 1956). Saccharification efficiency was determined as the ratio of sugars released by enzymatic hydrolysis to the amount of sugars present in the cell wall material before enzymatic hydrolysis. The solubilized sugar yield through enzymatic hydrolysis was calculated by the formula: solubilized sugar yield (g/plant) = cell wall carbohydrate yield of switchgrass biomass (g/plant) × saccharification efficiency.

### Statistical analysis

Triplicate samples were collected for each transgenic line. Data from each trait were subjected to analysis of variance (ANOVA). The significance of treatments was tested at the *P* < 0.05 level. Standard errors are provided in all tables and figures as appropriate. All the statistical analyses were performed using the SPSS package (SPSS Inc., Chicago, IL).

## Supporting Information

**Figure S1** Gene construct and Southern hybridization analysis of transgenic switchgrass plants.

**Figure S2** Phylogenetic analysis of the SPL family.

**Figure S3** Transcript abundance of putativeswitchgrass *SPLs* in different organs of wild-type switchgrass plants. Inflorescence, internode, leaf sheath and leaf blade were collected at R1 stage.

**Figure S4** AcBr lignin content of transgenic switchgrass biomass.

**Table S1** Primers used for this study.

**Table S2** Microarray analysis of the transgenic line T-44.

## References

[b1] Abramson M, Shoseyov O, Shani Z (2010). Plant cell wall reconstruction toward improved lignocellulosic production and processability. Plant Sci.

[b2] Alexandrova KS, Denchev PD, Conger BV (1996). Micropropagation of switchgrass by node culture. Crop Sci.

[b3] Biemelt S, Tschiersch H, Sonnewald U (2004). Impact of altered gibberellin metabolism on biomass accumulation, lignin biosynthesis, and photosynthesis in transgenic tobacco plants. Plant Physiol.

[b4] Bouton JH (2007). Molecular breeding of switchgrass for use as a biofuel crop. Curr. Opin. Genet. Dev.

[b5] Chen X (2009). Small RNAs and their roles in plant development. Annu. Rev. Cell Dev. Biol.

[b6] Chen F, Dixon RA (2007). Lignin modification improves fermentable sugar yields for biofuel production. Nat. Biotechnol.

[b7] Chuck G, Cigan AM, Saeteurn K, Hake S (2007). The heterochronic maize mutant *Corngrass1* results from overexpression of a tandem microRNA. Nat. Genet.

[b8] Doyle JJ, Doyle JL (1987). A rapid DNA isolation procedure for small quantities of fresh leaf tissue. Phytochem. Bull.

[b9] Dozmorov I, Centola M (2003). An associative analysis of gene expression array data. Bioinformatics.

[b10] Dubois M, Gilles KA, Hamilton JK, Rebers PA, Smith F (1956). Colorimetric method for determination of sugars and related substances. Anal. Chem.

[b11] Fu C, Mielenz JR, Xiao X, Ge Y, Hamilton C, Rodriguez M, Chen F, Foston M, Ragauskas A, Bouton J, Dixon RA, Wang Z-Y (2011a). Genetic manipulation of lignin reduces recalcitrance and improves ethanol production from switchgrass. Proc. Natl. Acad. Sci. U.S.A.

[b12] Fu C, Xiao X, Xi Y, Ge Y, Chen F, Bouton J, Dixon RA, Wang Z-Y (2011b). Downregulation of cinnamyl alcohol dehydrogenase (CAD) leads to improved saccharification efficiency in switchgrass. Bioenerg. Res.

[b13] Ge Y, Fu C, Bhandari H, Bouton J, Brummer EC, Wang Z-Y (2011). Pollen viability and longevity of switchgrass (Panicum virgatum L.). Crop Sci.

[b14] Gou J-Y, Felippes FF, Liu C-J, Weigel D, Wang J-W (2011). Negative regulation of anthocyanin biosynthesis in Arabidopsis by a miR156-targeted SPL transcription factor. Plant Cell Online.

[b15] Guretzky J, Biermacher J, Cook B, Kering M, Mosali J (2011). Switchgrass for forage and bioenergy: harvest and nitrogen rate effects on biomass yields and nutrient composition. Plant Soil.

[b16] Hatfield RD, Grabber J, Ralph J, Brei K (1999). Using the acetyl bromide assay to determine lignin concentrations in herbaceous plants: some cautionary notes. J. Agric. Food. Chem.

[b17] Henry RJ (2010). Evaluation of plant biomass resources available for replacement of fossil oil. Plant Biotechnol. J.

[b18] Hisano H, Nandakumar R, Wang Z-Y (2009). Genetic modification of lignin biosynthesis for improved biofuel production. In Vitro Cell. Dev. Biol.-Plant.

[b19] Jakob K, Zhou F, Paterson A (2009). Genetic improvement of C4 grasses as cellulosic biofuel feedstocks. In Vitro Cell. Dev. Biol.-Plant.

[b20] Jensen CS, Salchert K, Gao C, Andersen C, Didion T, Nielsen KK (2004). Floral inhibition in red fescue (*Festuca rubra* L.) through expression of a heterologous flowering repressor from *Lolium*. Mol. Breed.

[b21] Jin S, Kanagaraj A, Verma D, Lange T, Daniell H (2011). Release of hormones from conjugates: chloroplast expression of β-glucosidase results in elevated phytohormone levels associated with significant increase in biomass and protection from aphids or whiteflies conferred by sucrose esters. Plant Physiol.

[b22] Kebeish R, Niessen M, Thiruveedhi K, Bari R, Hirsch H-J, Rosenkranz R, Stabler N, Schonfeld B, Kreuzaler F, Peterhansel C (2007). Chloroplastic photorespiratory bypass increases photosynthesis and biomass production in Arabidopsis thaliana. Nat. Biotechnol.

[b23] Koslowsky S, Riegler H, Bergmüller E, Zrenner R (2008). Higher biomass accumulation by increasing phosphoribosylpyrophosphate synthetase activity in Arabidopsis thaliana and Nicotiana tabacum. Plant Biotechnol. J.

[b24] Kumar S, Tamura K, Nei M (2004). MEGA3: integrated software for molecular evolutionary genetics analysis and sequence alignment. Brief. Bioinforma.

[b25] Li R, Qu R (2011). High throughput *Agrobacterium*-mediated switchgrass transformation. Biomass Bioenerg.

[b26] Mann DGJ, LaFayette PR, Abercrombie LL, King ZR, Mazarei M, Halter MC, Poovaiah CR, Baxter H, Shen H, Dixon RA, Parrott WA, Stewart CN (2011). Gateway-compatible vectors for high-throughput gene functional analysis in switchgrass (Panicum virgatum L.) and other monocot species. Plant Biotechnol. J.

[b27] Matts J, Jagadeeswaran G, Roe BA, Sunkar R (2010). Identification of microRNAs and their targets in switchgrass, a model biofuel plant species. J. Plant Physiol.

[b28] McLaughlin SB, Kiniry JR, Taliaferro CM, De La Torre Ugarte D, Donald LS (2006). Projecting yield and utilization potential of switchgrass as an energy crop. Adv. Agron.

[b29] Moore KJ, Moser LE, Vogel KP, Waller SS, Johnson BE, Pedersen JF (1991). Describing and quantifying growth stages of perennial forage grasses. Agron. J.

[b30] Park W, Li J, Song R, Messing J, Chen X (2002). CARPEL FACTORY, a dicer homolog, and HEN1, a novel protein, act in microRNA metabolism in Arabidopsis thaliana. Curr. Biol.

[b31] Poethig RS (2009). Small RNAs and developmental timing in plants. Curr. Opin. Genet. Dev.

[b32] Rhoades MW, Reinhart BJ, Lim LP, Burge CB, Bartel B, Bartel DP (2002). Prediction of plant MicroRNA targets. Cell.

[b33] Saathoff AJ, Sarath G, Chow EK, Dien BS, Tobias CM (2011). Downregulation of cinnamyl-alcohol dehydrogenase in switchgrass by RNA silencing results in enhanced glucose release after cellulase treatment. PLoS One.

[b34] Sakamoto T, Morinaka Y, Ohnishi T, Sunohara H, Fujioka S, Ueguchi-Tanaka M, Mizutani M, Sakata K, Takatsuto S, Yoshida S, Tanaka H, Kitano H, Matsuoka M (2006). Erect leaves caused by brassinosteroid deficiency increase biomass production and grain yield in rice. Nat. Biotechnol.

[b35] Schmer MR, Vogel KP, Mitchell RB, Perrin RK (2008). Net energy of cellulosic ethanol from switchgrass. Proc. Natl. Acad. Sci. U.S.A.

[b36] Schwab R, Palatnik JF, Riester M, Schommer C, Schmid M, Weigel D (2005). Specific effects of MicroRNAs on the plant transcriptome. Dev. Cell.

[b37] Schwarz S, Grande A, Bujdoso N, Saedler H, Huijser P (2008). The microRNA regulated SBP-box genes *SPL9* and *SPL15* control shoot maturation in Arabidopsis. Plant Mol. Biol.

[b38] Shikata M, Koyama T, Mitsuda N, Ohme-Takagi M (2009). Arabidopsis SBP-Box genes *SPL10**SPL11* and *SPL2* control morphological change in association with shoot maturation in the reproductive phase. Plant Cell Physiol.

[b39] Somerville C, Youngs H, Taylor C, Davis SC, Long SP (2010). Feedstocks for lignocellulosic biofuels. Science.

[b40] Spangenberg G, Wang Z-Y, Potrykus I (1998). Biotechnology in Forage and Turf Grass Improvement.

[b41] Stewart CN (2007). Biofuels and biocontainment. Nat. Biotech.

[b42] Strauss SH, Kershen DL, Bouton JH, Redick TP, Tan H, Sedjo RA (2010). Far-reaching deleterious impacts of regulations on research and environmental studies of recombinant DNA-modified perennial biofuel crops in the United States. Bioscience.

[b43] Varkonyi-Gasic E, Wu R, Wood M, Walton E, Hellens R (2007). Protocol: a highly sensitive RT-PCR method for detection and quantification of microRNAs. Plant Methods.

[b44] Wang Z-Y, Ge Y (2006). Recent advances in genetic transformation of forage and turf grasses. In Vitro Cell. Dev. Biol.-Plant.

[b45] Wang J-W, Czech B, Weigel D (2009). miR156-regulated SPL transcription factors define an endogenous flowering pathway in Arabidopsis thaliana. Cell.

[b46] Wang H, Avci U, Nakashima J, Hahn MG, Chen F, Dixon RA (2010). Mutation of WRKY transcription factors initiates pith secondary wall formation and increases stem biomass in dicotyledonous plants. Proc. Natl. Acad. Sci. U.S.A.

[b47] Wu G, Park MY, Conway SR, Wang J-W, Weigel D, Poethig RS (2009). The sequential action of miR156 and miR172 regulates developmental timing in Arabidopsis. Cell.

[b48] Xi Y, Fu C, Ge Y, Nandakumar R, Hisano H, Bouton J, Wang Z-Y (2009). *Agrobacterium*-mediated transformation of switchgrass and inheritance of the transgenes. Bioenerg. Res.

[b49] Xie K, Wu Can, Xiong L (2006). Genomic organization, differential expression, and interaction of SQUAMOSA Promoter-Binding-Like tanscription factors and microRNA156 in rice. Plant Physiol.

[b50] Xing S, Salinas M, Hohmann S, Berndtgen R, Huijser P (2010). miR156-targeted and nontargeted SBP-Box transcription factors act in concert to secure male fertility in Arabidopsis. Plant Cell.

[b51] Xu B, Escamilla-Treviño LL, Sathitsuksanoh N, Shen Z, Shen H, Zhang YHP, Dixon RA, Zhao B (2011). Silencing of 4-coumarate:coenzyme A ligase in switchgrass leads to reduced lignin content and improved fermentable sugar yields for biofuel production. New Phytol.

[b52] Yuan JS, Tiller KH, Al-Ahmad H, Stewart NR, Stewart CN (2008). Plants to power: bioenergy to fuel the future. Trends Plant Sci.

[b53] Zhang B, Pan X, Cobb GP, Anderson TA (2006). Plant microRNA: a small regulatory molecule with big impact. Dev. Biol.

