# Supplementary material for: Overexpression of miR156 in switchgrass (Panicum virgatum L.) results in various morphological alterations and leads to improved biomass production
Source: Plant Biotechnol J. 2012 May;10(4):443–52. doi: 10.1111/j.1467-7652.2011.00677.x (PMC3489066; doi:10.1111/j.1467-7652.2011.00677.x)
Supplement: Supplementary file 1 [file pbi0010-0443-SD1.docx]

**Supporting information**

**Fig. S1** Gene construct and Southern hybridization analysis of transgenic switchgrass plants.

(a) Schematic map of the expression cassette within the T-DNA border sequences of the pANIC6A-Pre-OsmiRNA156b construct used for genetic transformation of switchgrass. (b) Southern blot hybridization of a DNA blot containing *Nco*I-digested genomic DNA isolated from regenerated switchgrass plants and hybridized with the *hph* probe. Ctrl: non-transformed plant serving as control.

**Fig. S2** Phylogenetic analysis of the SPL family. Rice SPL protein sequences were downloaded from the Rice Genome Annotation Project Database (http://rice.plantbiology.msu.edu/). The OsSPLs accession numbers are: LOC_*Os01g18850* (OsSPL1), LOC_Os01g69830 (OsSPL2), LOC_Os02g04680 (OsSPL3), LOC_Os02g07780 (OsSPL4), LOC_Os02g08070 (OsSPL5), LOC_Os03g61760 (OsSPL6), LOC_Os04g46580 (OsSPL7), LOC_Os04g56170 (OsSPL8), LOC_Os05g33810 (OsSPL9), LOC_Os06g44860 (OsSPL10), LOC_Os06g45310 (OsSPL11), LOC_Os06g49010 (OsSPL12), LOC_Os07g32170 (OsSPL13), LOC_Os08g39890 (OsSPL14), LOC_Os08g40260 (OsSPL15), LOC_Os08g41940 (OsSPL16), LOC_Os09g31438 (OsSPL17), LOC_Os09g32944 (OsSPL18), and LOC_Os011g30370 (OsSPL19). Switchgrass *SPL* genes sequences were downloaded from the Switchgrass Unique Transcript Sequences Database (http://switchgrassgenomics.noble.org/). The *PvSPL* genes accession numbers are: AP13ITG60657 (*PvSPL1*), AP13CTG29191 (*PvSPL2*), AP13ITG56500 (*PvSPL3*), KanlowCTG20060 (*PvSPL4*), KanlowSGLT49238 (*PvSPL5*), KanlowCTG31732 (*PvSPL6*), KanlowCTG41639 (*PvSPL7*), KanlowCTG07384 (*PvSPL8*). Their corresponding protein sequences were predicted by ORF-finder tool on the NCBI website. The SPL genes marked by circle, triangle and square were selected for further analysis by quantitative RT-PCR.

**Fig. S3** Transcript abundance of putative switchgrass *SPLs* in different organs of wild-type switchgrass plants. Inflorescence, internode, leaf sheath and leaf blade were collected at R1 stage. The abundance of *PvSPL1*, *2*, *3* and *6* transcripts was determined by quantitative RT-PCR. Switchgrass *Ubq1* was used as the reference for normalization.

**Fig. S4** AcBr lignin content of transgenic switchgrass biomass. The transgenic and control plants were harvested after 6-month growth in the greenhouse. CWR: cell wall residue. Values are means ± SE (n=3). One or two asterisks indicate significance corresponding to P < 0.05 or 0.01 (One way ANOVA, Dunnett’s test).
