# Supplementary figures and images for: Overexpression of miR156 in switchgrass (Panicum virgatum L.) results in various morphological alterations and leads to improved biomass production
Source: Plant Biotechnol J. 2012 May;10(4):443–52. doi: 10.1111/j.1467-7652.2011.00677.x (PMC3489066; doi:10.1111/j.1467-7652.2011.00677.x)

**(a)**

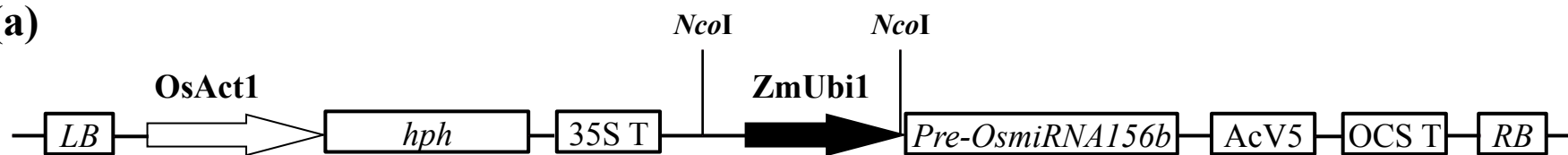

**(b)**

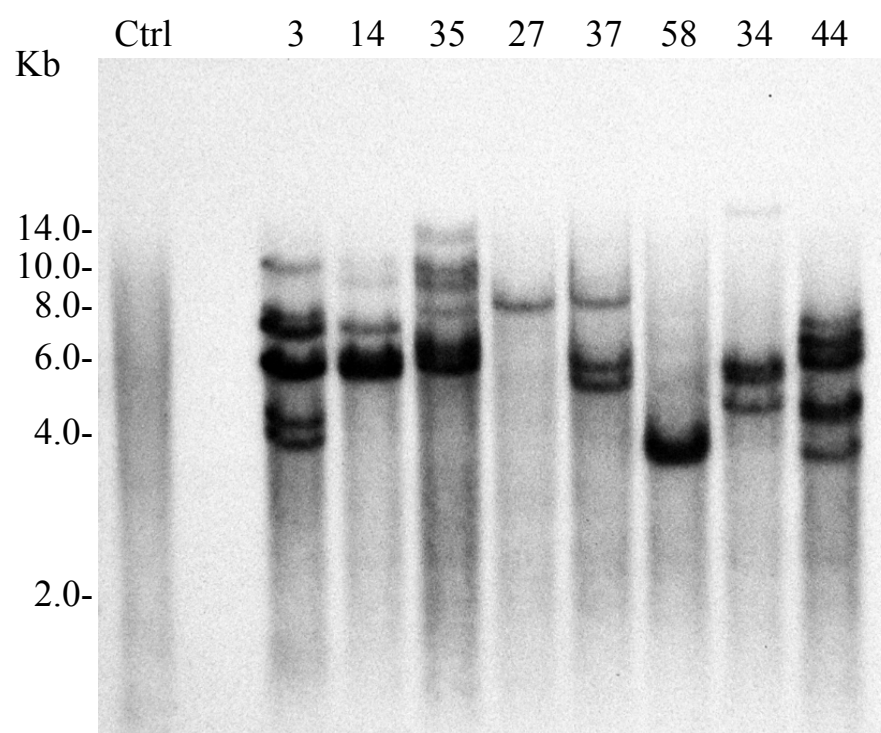

Supplement: Supplementary file 2 [file pbi0010-0443-SD2.pdf]

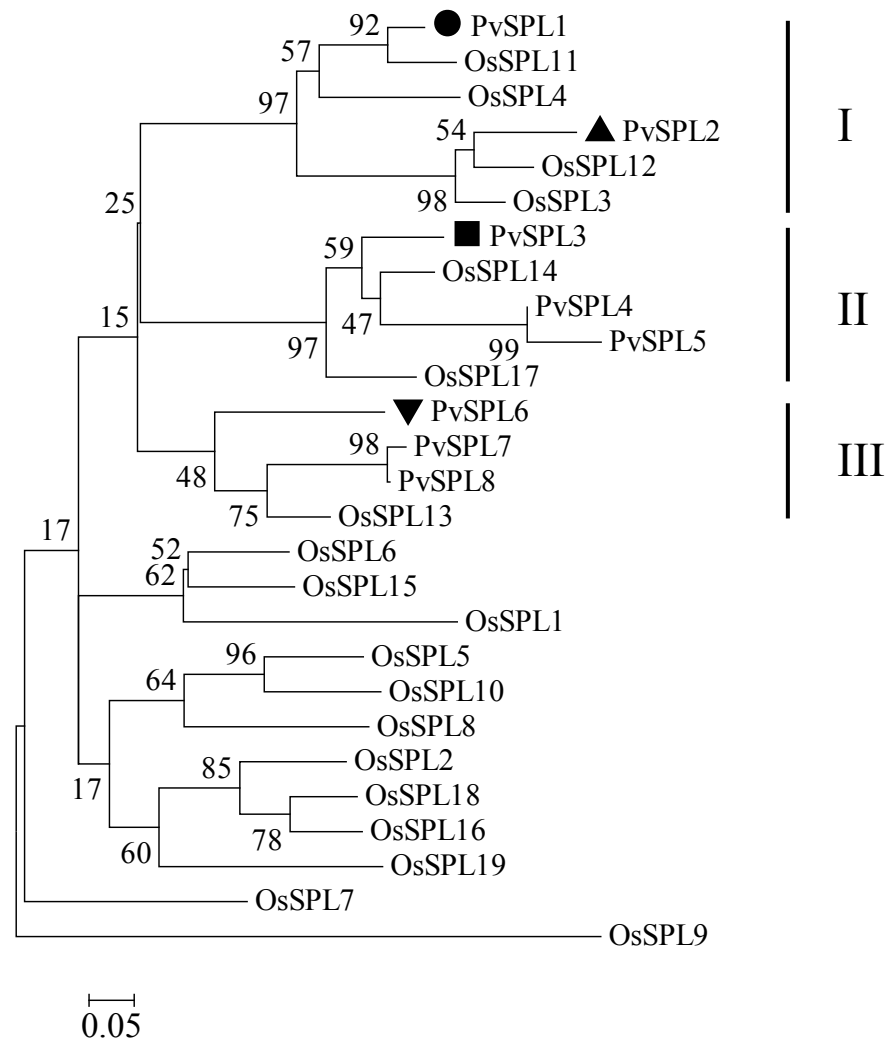

Supplement: Supplementary file 3 [file pbi0010-0443-SD3.pdf]

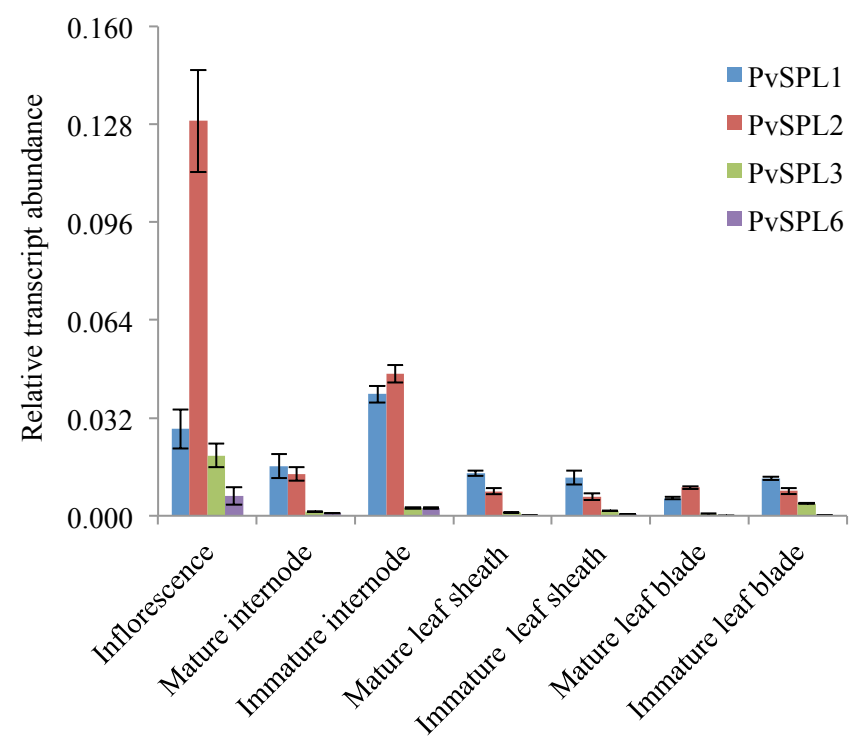

Supplement: Supplementary file 4 [file pbi0010-0443-SD4.pdf]

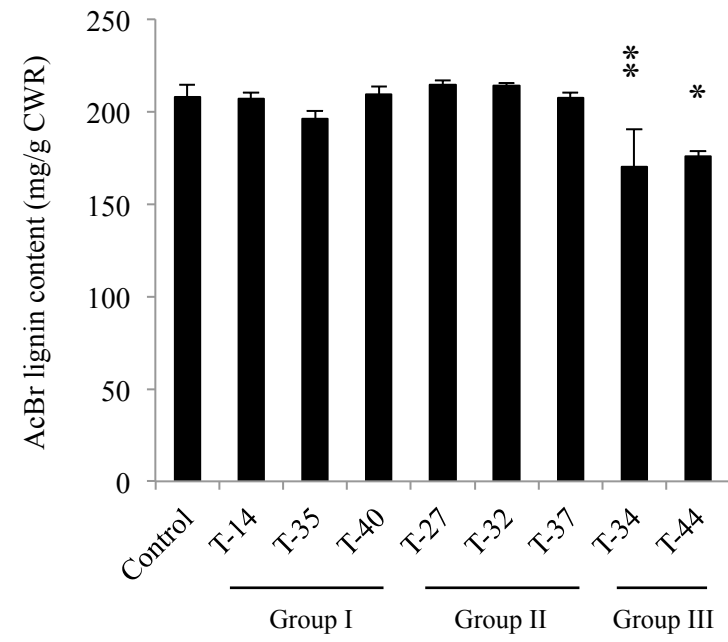

Supplement: Supplementary file 5 [file pbi0010-0443-SD5.pdf]
