# Supplementary material for: Overexpression of miR156 in switchgrass (Panicum virgatum L.) results in various morphological alterations and leads to improved biomass production
Source: Plant Biotechnol J. 2012 May;10(4):443–52. doi: 10.1111/j.1467-7652.2011.00677.x (PMC3489066; doi:10.1111/j.1467-7652.2011.00677.x)
Supplement: Supplementary file 6 [file pbi0010-0443-SD6.docx]

Table S1 Primers used for this study.

| Gene name | Primer sequences |
| --- | --- |
| *hph3* | AAGGAATCGGTCAATACACTACATGG |
| *hph4* | AAGACCAATGCGGAGCATATACG |
| Pre-miR156-F | CACCACAGTTTAATTTATTTCTTGG |
| Pre-miR156-R | CTAGGCAGAAAATTTAACAGGAG |
| qmiRNAUniversal-R | GTGCAGGGTCCGAGGT |
| qmiR156-RT | GTCGTATCCAGTGCAGGGTCCGAGGTATTCGCACTGGATACGACGTGCTC |
| qmiR156-F | GCGGCGGTGACAGAAGAGAGT |
| qmiR390-RT | GTCGTATCCAGTGCAGGGTCCGAGGTATTCGCACTGGATACGACGGCGCT |
| qmiR390-F | TCGCTAAGCTCAGGAGGGAT |
| *PvSPL1*-F | GATGGCCTGGGGTCTTG |
| *PvSPL1*-R | TGGCCTACGCTCAGTTC |
| *PvSPL2*-F | GCGCGGTTTCAGGCTCTCG |
| *PvSPL2*-R | CCTCGCAACCGGACAATGGA |
| *PvSPL3*-F | GCCACGCCACGACCACGAGAC |
| *PvSPL3*-R | CGGGCTACACGGGAAAGGGGAACT |
| *PvSPL6*-F | GCAGCGGCGGCGACCAGGAC |
| *PvSPL6*-R | GGCAGATCGACAGACACAGCACAC |
| *PvUbiquitin*-F | TTCGTGGTGGCCAGTAAG |
| *PvUbiquitin*-R | AGAGACCAGAAGACCCAGGTACAG |

Table S2 Microarray analysis of the transgenic line T-44.

| **A: Probe sets with expression level upregulated more than 5-fold in T-44** | | |
| --- | --- | --- |
| **Probe set** | **Annotation** | **T-44/Ctrl** |
| AP13CTG22802_s_at | cd04660/nsLTP_like: Non-specific lipid-transfer protein (nsLTP)-like subfamily./0.000000000000009 | 64.7 |
| OTHSWSLT28307_s_at | pfam01419/Jacalin-like lectin domain./0.0000008 | 59.1 |
| AP13ITG42062_s_at | PHA03247/large tegument protein UL36./0.000000000000001 | 40.1 |
| AP13ITG53035_at | pfam03059/Nicotianamine synthase protein./4E-138 | 23.7 |
| AP13CTG30873_at | pfam00635/MSP (Major sperm protein) domain. /0.00000002 | 22.6 |
| AP13ITG70317_at | pfam00305/Lipoxygenase./0 | 18.7 |
| AP13CTG28994_s_at | pfam06830/Root cap./6E-28 | 16.1 |
| AP13CTG22802_at | cd04660/nsLTP_like: Non-specific lipid-transfer protein (nsLTP)-like subfamily./0.000000000000009 | 15.5 |
| AP13CTG05855_x_at | pfam00232/Glycosyl hydrolase family 1./6E-103 | 14.3 |
| AP13CTG15489_at | PHA03245/large tegument protein UL36./0.000000000000008 | 14.0 |
| OTHSWSLT23951_s_at | pfam00232/Glycosyl hydrolase family 1./7E-45 | 13.4 |
| AP13ITG55117_at | PHA03245/large tegument protein UL36./6E-22 | 13.1 |
| OTHSWCTG07474_at | cd00684/Plant Terpene Cyclases, Class 1. /2E-67 | 12.7 |
| AP13CTG14748_at | PHA03245/large tegument protein UL36. /0.009 | 11.3 |
| AP13CTG59158_s_at | pfam00280/Potato inhibitor I family./4E-27 | 9.3 |
| AP13ITG67654_at | PLN02992/coniferyl-alcohol glucosyltransferase./4E-64 | 8.8 |
| AP13CTG17100_s_at | pfam07712/Stress up-regulated Nod 19./3E-162 | 8.7 |
| OTHSWCTG28881_at | PLN02441/cytokinin dehydrogenase./4E-22 | 8.6 |
| AP13CTG17100_at | pfam07712/Stress up-regulated Nod 19./3E-162 | 8.3 |
| KanlowCTG22980_s_at | pfam00232/Glycosyl hydrolase family 1./3E-60 | 8.2 |
| AP13CTG17601_at | PHA03307/transcriptional regulator ICP4./0.0002 | 7.9 |
| AP13ITG39255_s_at | pfam06830/Root cap. /6E-27 | 7.9 |
| AP13CTG15472_s_at | pfam03134/TB2/DP1, HVA22 family. /1E-29 | 7.7 |
| AP13CTG50688_at | smart00220/Serine/Threonine protein kinases, catalytic domain./2E-19 | 7.3 |
| AP13CTG50688_s_at | smart00220/Serine/Threonine protein kinases, catalytic domain. /2E-19 | 7.3 |
| KanlowCTG25218_at | pfam11145/Protein of unknown function (DUF2921)./5E-48 | 7.2 |
| AP13CTG22460_at | cd04660/nsLTP_like: Non-specific lipid-transfer protein (nsLTP)-like subfamily./0.00000000000005 | 7.0 |
| AP13ITG60690_at | PRK12678/transcription termination factor Rho; Provisional/0.000000002 | 6.9 |
| AP13ITG73929_s_at | TIGR02189/Glutaredoxin-like family. /2E-34 | 6.8 |
| OTHSWCTG11457_at | cd00684/Plant Terpene Cyclases, Class 1../9E-47 | 6.8 |
| KanlowCTG41234_at | pfam00305/Lipoxygenase./9E-168 | 6.7 |
| AP13ITG69979_at | PLN02299/aminocyclopropanecarboxylate oxidase./0 | 6.6 |
| KanlowCTG08413_at | PLN03007/UDP-glucosyltransferase family protein./9E-58 | 6.4 |
| AP13CTG31838_at | pfam00067/Cytochrome P450./9E-35 | 6.3 |
| KanlowCTG24566_at | pfam02496/ABA/WDS induced protein./1E-21 | 6.3 |
| AP13ITG69078_at | PLN02242/methionine gamma-lyase./0 | 6.1 |
| KanlowCTG18829_at | pfam03350/Uncharacterized protein family./2E-27 | 6.0 |
| AP13.12748.m00004_s_at | pfam00854/POT family./1E-61 | 5.9 |
| AP13CTG23436_s_at | PLN02687/flavonoid 3'-monooxygenase./3E-127 | 5.9 |
| AP13ITG64948_at | PRK12678/transcription termination factor Rho./0.0000000008 | 5.8 |
| AP13CTG04892_s_at | smart00338/basic region leucin zipper./0.00000002 | 5.8 |
| AP13CTG24565_at | pfam00854/POT family./2E-76 | 5.7 |
| AP13CTG16311_at | cd03244/Domain 2 of the ABC subfamily C./4E-90 | 5.6 |
| AP13ITG38938_s_at | COG1253/Hemolysins and related proteins containing CBS domains./0.0006 | 5.4 |
| AP13CTG15593_s_at | PLN02290/cytokinin trans-hydroxylase./4E-84 | 5.4 |
| AP13ITG66196_at | smart00380/DNA-binding domain in plant proteins such as APETALA2 and EREBPs./1E-23 | 5.4 |
| AP13CTG31838_s_at | pfam00067/Cytochrome P450./9E-35 | 5.4 |
| AP13CTG23185RC_at | cd00322/Ferredoxin reductase (FNR), an FAD and NAD(P) binding protein. /0.0000000000000003 | 5.3 |
| AP13CTG12974_s_at | pfam01490/Transmembrane amino acid transporter protein./6E-33 | 5.3 |
| AP13CTG17251_s_at | PRK00913/leucyl aminopeptidase./1E-144 | 5.3 |
| AP13ITG75808_at | PLN02670/transferase, transferring glycosyl groups./3E-22 | 5.2 |
| AP13CTG25576_s_at | cd00170/Sec14p-like lipid-binding domain./0.0000001 | 5.2 |
| AP13CTG30643_at | PLN02248/cellulose synthase./2E-28 | 5.2 |
| AP13CTG06479_at | pfam03350/Uncharacterized protein family./2E-34 | 5.2 |
| AlamoCTG02653_x_at | PLN02578/hydrolase./0.000000000005 | 5.1 |
| AP13ITG39191_s_at | pfam01490/Transmembrane amino acid transporter protein./5E-65 | 5.1 |
| AP13CTG27921_at | pfam06830/Root cap./1E-26 | 5.1 |
| AP13CTG14851_at | pfam00067/Cytochrome P450./1E-48 | 5.1 |
| AP13ITG58535_at | pfam00332/Glycosyl hydrolases family 17./2E-121 | 5.0 |
| AP13ITG60472_s_at | pfam07172/Glycine rich protein family./0.000000000000006 | 5.0 |
| AP13ITG52053_s_at | PLN02998/hydrolase, hydrolyzing O-glycosyl compounds/glucosidase./2E-39 | 5.0 |
| AP13CTG11976_s_at | PLN02687/flavonoid 3'-monooxygenase./6E-95 | 5.0 |
| AP13ITG62412_s_at | unknown | 12.7 |
| AP13ITG62412_at | unknown | 11.0 |
| AP13ITG65548_at | unknown | 9.1 |
| KanlowCTG23007_at | unknown | 9.1 |
| AlamoCTG00456_s_at | unknown | 9.1 |
| AP13CTG54404_at | unknown | 8.0 |
| VS16ITG09542_at | unknown | 7.9 |
| KanlowCTG28441_at | unknown | 7.9 |
| AP13CTG17395_s_at | unknown | 7.2 |
| KanlowCTG21021_at | unknown | 7.0 |
| VS16ITG20386_s_at | unknown | 6.4 |
| AP13CTG15662_at | unknown | 6.3 |
| OTHSWCTG18066_at | unknown | 6.1 |
| KanlowSLT55014_s_at | unknown | 5.9 |
| AP13ITG77544_at | unknown | 5.9 |
| AP13CTG16179_at | unknown | 5.8 |
| AP13ITG55907_at | unknown | 5.5 |
| AP13ITG64528_at | unknown | 5.2 |
| AP13ITG59493_s_at | unknown | 5.2 |
| AP13ITG59493_at | unknown | 5.1 |
| **B: Probe sets with expression level downregulated more than 5-fold in T-44** | | |
| **Probe set** | **Annotation** | **T0-44/Ctrl** |
| AP13CTG15220_at | PLN02395/glutathione transferase/9E-44 | 0.17 |
| KanlowCTG08970_at | pfam01255/Putative undecaprenyl diphosphate synthase./3E-70 | 0.19 |
| AP13ITG60148_at | PLN02395/glutathione transferase/2E-43 | 0.18 |
| AP13CTG17241_at | PRK07003/DNA polymerase III subunits gamma and tau./0.00000001 | 0.18 |
| AP13CTG10505_at | TIGR00393/KpsF/GutQ family protein./2E-55 | 0.18 |
| AP13CTG17686_s_at | pfam02209/Villin headpiece domain./0.0000000002 | 0.18 |
| AP13ITG56786_at | pfam07911/Protein of unknown function (DUF1677). /1E-26 | 0.18 |
| OTHSWCTG18065_at | pfam02902/Ulp1 protease family, C-terminal catalytic domain./0.00003 | 0.17 |
| AP13CTG16136_s_at | smart00835/Cupin./2E-31 | 0.17 |
| AP13CTG15515_at | cd04852/Peptidase S8 family domain, uncharacterized subfamily 3./3E-26 | 0.17 |
| AP13ITG64213_at | cd05472/Chloroplast Nucleoids DNA-binding Protease, catalyzes the degradation of ribulose-1,5-bisphosphate carboxylase/oxygenase./7E-59 | 0.17 |
| AP13CTG02748_at | PLN02395/glutathione transferase/2E-45 | 0.16 |
| AP13ITG50736_s_at | cd03187/GST_C family, Class Phi subfamily./0.001 | 0.16 |
| AP13CTG26715_s_at | PLN02395/glutathione transferase/1E-43 | 0.15 |
| KanlowCTG15508_at | pfam01190/Pollen proteins Ole e I like./0.000000000000002 | 0.14 |
| AP13CTG02940_at | PLN02279/ent-kaurene synthase/1E-166 | 0.03 |
| AP13CTG17686_at | pfam02209/Villin headpiece domain./0.0000000002 | 0.15 |
| AP13CTG55563_at | cd01958/HPS_like: Hydrophobic Protein from Soybean (HPS)-like subfamily. /3E-23 | 0.15 |
| AP13CTG24870_s_at | PLN02316/synthase/transferase/0 | 0.13 |
| AP13CTG05770_at | COG0071/Molecular chaperone (small heat shock protein) [Posttranslational modification, protein turnover, chaperones]/0.000004 | 0.17 |
| AlamoCTG12662_at | smart00614/BED zinc finger. DNA-binding domain in chromatin-boundary-element-binding proteins and transposases/0.0006 | 0.19 |
| AP13ITG37289_s_at | cd00265/MEF2 (myocyte enhancer factor 2)-like/Type II subfamily of MADS ( MCM1, Agamous, Deficiens, and SRF (serum response factor) box family of eukaryotic transcriptional regulators./7E-33 | 0.15 |
| AP13ITG69824RC_at | COG0724/RNA-binding proteins (RRM domain) [General function prediction only]/8E-20 | 0.10 |
| KanlowCTG20060_s_at | pfam03110/SBP domain. SBP domains (for SQUAMOSA-pROMOTER BINDING PROTEIN) are found in plant proteins./7E-41 | 0.16 |
| KanlowCTG07384_s_at | pfam03110/SBP domain. SBP domains (for SQUAMOSA-pROMOTER BINDING PROTEIN) are found in plant proteins./3E-38 | 0.13 |
| OTHSWCTG15562_s_at | Unknown | 0.09 |
| KanlowCTG45182_at | Unknown | 0.18 |
| AP13CTG51007_s_at | Unknown | 0.18 |
| AP13CTG09187_s_at | Unknown | 0.17 |
| AP13CTG12699_at | Unknown | 0.17 |
| KanlowCTG28863_at | Unknown | 0.17 |
| OTHSWCTG18057_s_at | Unknown | 0.16 |
| OTHSWCTG13117_at | Unknown | 0.16 |
| AP13CTG22347_s_at | Unknown | 0.16 |
| AP13ITG66066_at | Unknown | 0.15 |
| OTHSWCTG17776_s_at | Unknown | 0.13 |
| KanlowCTG40576_s_at | Unknown | 0.13 |
| KanlowCTG41507_s_at | Unknown | 0.08 |
| KanlowCTG41507_at | Unknown | 0.05 |
| **C: Putative *SPL* probe sets with expression level downregulated more than 2-fold in T-44** | | |
| **Probe set** | **Annotation (SPL genes)** | **T-44/Ctrl** |
| KanlowCTG07384_s_at | pfam03110/SBP domain. SBP domains (for SQUAMOSA-pROMOTER BINDING  PROTEIN) are found in plant proteins./3E-38 | 0.13 |
| KanlowCTG20060_s_at | pfam03110/SBP domain. SBP domains (for SQUAMOSA-pROMOTER BINDING PROTEIN) are found in plant proteins./7E-41 | 0.16 |
| AP13CTG29191_at | pfam03110/SBP domain. SBP domains (for SQUAMOSA-pROMOTER BINDING PROTEIN) are found in plant proteins./2E-42 | 0.26 |
| AP13ITG60657_at | pfam03110/SBP domain. SBP domains (for SQUAMOSA-pROMOTER BINDING PROTEIN) are found in plant proteins./2E-41 | 0.24 |
| AP13ITG56500_at | pfam03110/SBP domain. SBP domains (for SQUAMOSA-pROMOTER BINDING PROTEIN) are found in plant proteins./1E-39 | 0.34 |
| KanlowCTG41639_s_at | pfam03110/SBP domain. SBP domains (for SQUAMOSA-pROMOTER BINDING PROTEIN) are found in plant proteins./5E-38 | 0.21 |
| KanlowCTG49238_s_at | pfam03110/SBP domain. SBP domains (for SQUAMOSA-pROMOTER BINDING PROTEIN) are found in plant proteins./0.000000000004 | 0.34 |
| KanlowCTG31732_s_at | pfam03110/SBP domain. SBP domains (for SQUAMOSA-pROMOTER BINDING PROTEIN) are found in plant proteins./2E-34 | 0.30 |
